# The Relationship between the Antioxidant System and Proline Metabolism in the Leaves of Cucumber Plants Acclimated to Salt Stress

**DOI:** 10.3390/cells10030609

**Published:** 2021-03-10

**Authors:** Marcin Naliwajski, Maria Skłodowska

**Affiliations:** Department of Plant Physiology and Biochemistry, Faculty of Biology and Environmental Protection, University of Lodz, ul. Banacha 12/16, 90-237 Lodz, Poland; maria.sklodowska@biol.uni.lodz.pl

**Keywords:** *Cucumis sativus*, salt stress, acclimation, antioxidant enzymes, proline metabolism

## Abstract

The study examines the effect of acclimation on the antioxidant system and proline metabolism in cucumber leaves subjected to 100 and 150 NaCl stress. The levels of protein carbonyl group, thiobarbituric acid reactive substances, α-tocopherol, and activity of ascorbate and glutathione peroxidases, catalase, glutathione *S*-transferase, pyrroline-5-carboxylate: synthetase and reductase as well as proline dehydrogenase were determined after 24 and 72 h periods of salt stress in the acclimated and non-acclimated plants. Although both groups of plants showed high α-tocopherol levels, in acclimated plants was observed higher constitutive concentration of these compounds as well as after salt treatment. Furthermore, the activity of enzymatic antioxidants grew in response to salt stress, mainly in the acclimated plants. In the acclimated plants, protein carbonyl group levels collapsed on a constitutive level and in response to salt stress. Although both groups of plants showed a decrease in proline dehydrogenase activity, they differed with regard to the range and time. Differences in response to salt stress between the acclimated and non-acclimated plants may suggest a relationship between increased tolerance in acclimated plants and raised activity of antioxidant enzymes, high-level of α-tocopherol as well, as decrease enzyme activity incorporates in proline catabolism.

## 1. Introduction

Soil salinity, caused by inter alia salt pollution, is a serious global problem due to the ionic and osmotic stresses it places on in plants [1]. Soils are classified as saline when the electrical conductivity (EC) exceeds 4 dS m^−1^ for a saturated soil solution (approximately 40 mM NaCl) and 15 dS m^−1^ for soil [2]. The UN Food and Agriculture Organization estimates that the saline soils constitute 397 million ha worldwide and sodic soils 434 million ha [3].

Plants can acquire tolerance to salt stress by adapting their metabolism to protect major cellular processes such as photosynthesis and respiration [4]. These mechanisms act synergistically through altering the metabolisms and activating new biochemical pathways; they can also influence the synthesis of chemical compounds which may have been absent or present at low concentrations before the occurrence of stress [5,6,7].

Due to the greater prevalence of salt pollution and corresponding increases in NaCl concentration in soils, there is a need to obtain a glycophyte that could function properly and reproduce under salt stress. The acclimation to salinity is one of the methods by which plants can become NaCl stress-resistant. Such acclimation can take place in few ways, but two popular approaches involve treating seedlings with a low concentration of sodium chloride or the selection of stress-adapted tissue and/or cell cultures [8,9]. The first studies showing an increase in plant resistance under the influence of acclimation were carried out by Strogonov [10]. Subsequent studies included the acclimation of cell and tissue cultures, followed by their selection, organogenesis and full regeneration of plants resistant to salt stress [11,12]. A suitable acclimation method may well reduce the agricultural losses caused by soil salinity. However, the whole acclimation process must first be clarified, from the molecular and biochemical through cellular and physiological to whole plants [8,11].

Oxidative stress is common in plants. Reactive oxygen species (ROS), such as singlet oxygen (^1^O_2_), superoxide anions (O_2_^.−^), hydrogen peroxide (H_2_O_2_) and hydroxyl radicals (HO^.^), are generated in the cells of all aerobic organisms under physiological conditions and their levels are controlled by the antioxidant system [13]. Under stressful conditions, their generation increases significantly, which induces changes in antioxidant potential. In recent years, it has become clear that ROS plays a dual role in plants, both as toxic compounds and key regulators of various biological processes such as growth, cell cycle, programmed cell death, hormone signaling, biotic and abiotic cell responses and development [13,14].

Due to their strong oxidizing properties, elevated ROS concentrations can cause irreversible or hardly reversible changes in cellular biomolecules such as proteins, lipids and nucleic acids, resulting in damage to cell and organelle membranes [15]. Due to them constituting over 50% of the dry weight of cells, proteins are the main target of the action of ROS. Protein oxidation is generally defined as the covalent modification of proteins induced by ROS and byproducts of pro-oxidative reactions. Among various types of oxidative protein modification, most are irreversible, and their products are considered as markers of oxidative stress [15,16]. Being common components of plant plasma membranes, polyunsaturated fatty acids are particularly exposed to the action of ROS, especially singlet oxygen and hydroxyl radicals. These react with polyunsaturated fatty acids, leading to the formation of lipid peroxides. The changes generated by ROS the physicochemical structure of the plasma membrane: it becomes less fluid, and its permeability increases, resulting in electrolyte leakage [15,17].

The adverse effects of ROS in cells are countered by a biologically complex and integrated system of antioxidant protection. Its components are divided into nonenzymatic ones, such as low molecular-weight phenolic compounds, glutathione, ascorbate, tocopherols and carotenoids, and enzymatic ones such as superoxide dismutases and catalase (CAT), as well as the peroxidases: ascorbate peroxidase (APX) and glutathione peroxidase (GPX) [13]. Peroxidases and catalase are mainly responsible for scavenging H_2_O_2_. Plant peroxidases activities are believed to occur through a range of diverse processes. Generally, peroxidases catalyze the redox reactions between H_2_O_2_ and various reductants, such as phenols, amines and alcohols, but they also are involved in lignin synthesis and the formation of the cross-links between cell wall components, which occurs during both normal plant growth and defense responses [18]. In the case of H_2_O_2_ metabolism, peroxidases play a dual role: while they can remove H_2_O_2_, they also catalyze various oxidative reactions leading to its generation [19,20]. The GPX family are antioxidant enzymes involved in scavenging H_2_O_2_, organic hydroperoxides and, like APX, in the regulation of the cellular redox homeostasis [20,21].

APX uses ascorbate to reduce H_2_O_2_ to water, with the concomitant generation of monodehydroascorbate. This enzyme family consists of at least five different isoforms: viz. thylakoid and glyoxysome membrane, stromal, cytosolic and apoplast. The H_2_O_2_ disproportionation reaction catalyzed by catalase is an example of a hydroperoxidation reaction, in which hydrogen peroxide is both a hydrogen donor and acceptor. This enzyme is mainly located in peroxisomes; being is less well established in other cell compartments [22]. Together with glutathione peroxidase, phospholipid hydroperoxide glutathione peroxidase and glutathione *S*-transferase (GST), the glutathione-dependent enzymes participate in the detoxification processes of lipid peroxides. GST is not a typical antioxidant enzyme, and unlike peroxidases, it is not able to reduce the hydrogen peroxide generated during oxidative stress. GST uses glutathione to create conjugates with extrinsic (herbicides, xenobiotics) and intrinsic electro- or nucleophilic compounds. The reaction catalyzed by GST binds these compounds to the sulfur group of glutathione. Glutathione *S*-transferase can also participate in the removal of products resulting from the reaction of ROS with cellular biomolecules, e.g., lipid peroxides. The glutathione *S*-conjugates formed in the GST-catalyzed reaction are metabolized to stable, nonreactive products stored in the vacuole [23].

Proline is a water-soluble imino acid classified as a compatible compound, i.e., it does not damage cell structures at high concentrations, but it does lower the cell osmotic potential. It was shown that the disintegration of plasmalemma observed during osmotic and drought stresses, caused, e.g., by denaturation of membrane proteins, is significantly limited in the cells of the plants with elevated proline content. Proline has a protective effect on phospholipids, plasmalemma, mitochondria and plastid membranes [8,24]. Proline has been found to contribute to the scavenging of hydroxyl radicals via a proline-proline cycle without consumption of proline, but it does not appear to quench singlet oxygen [25,26]. Our studies based on an identical experimental model found salt stress to cause an increase in proline concentration [27]. Salt exposure increased the proline level in the leaves of both acclimated and non-acclimated cucumber plants; in addition, the acclimated plants had lower levels of proline compared to the non-acclimated plants, both constitutively and after stress treatment. Since proline accumulation is a common metabolic response of higher plants to water deficit and salt stress [24], we hypothesize that the lower concentration of proline observed in the acclimated plants may result from its possible metabolism to proline betaine and hydroxyprolinebetaine, which are more potent osmoprotectants than proline.

In plant cells, glutamic acid acts as a substrate in the major pathway of proline biosynthesis, which is reduced by pyrroline-5-carboxylic acid synthetase (P5CS) to pyrroline-5-carboxylic acid (P5C) and then by pyrroline-5-carboxylic acid reductase (P5CR) to proline [28]. Proline dehydrogenase (PDH) is an enzyme that initiates the proline degradation pathway. PDH oxidizes proline to P5C, where FAD^+^ is used as an electron acceptor. In a further step, pyrroline-5-carboxylic acid dehydrogenase converts P5C to glutamate. This pathway is the only known pathway of proline catabolism to glutamate in higher plants so far. PDH is found in the mitochondria, where it is bound to their inner membrane [28,29]. The aim of the present study was to assess the ability of cucumber plants to acclimate to NaCl stress by evaluating changes in the proline and antioxidant metabolism associated with this process. To this end, the study examines a range of parameters in cucumber leaves taken from stressed plants which had been either acclimated or non-acclimated to salinity stress: APX, GPX, CAT, GST, P5CS, P5CR, PDH activity, and lipid peroxidation, carbonyl group content and α-tocopherol levels were measured 24 h and 72 h after being subjected to moderate and severe NaCl stress in the two groups of plants.

## 2. Material and Methods

### 2.1. Plant Material and Experimental Design

Cucumber seeds (*Cucumis sativus* L.) cv “Cezar” were germinated in Petri dishes for seven days in a controlled environment, i.e., a growth chamber, at a temperature of 23 ± 0.5 °C with 16 h light/8 h dark photoperiod at a light intensity of 150 μmol m^−2^ s^−1^ photon flux density and 60–70% relative humidity. The seedlings were planted into a peat-based substrate (commercial) containing plastic pots (400 cm^3^), one seedling per pot. According to the producer’s information: mineral nutrient content in 1 L of peat-based substrate: 80–120 mg N, 60–80 mg P, 160–220 mg K, 70–120 mg Mg, and 1500–2000 Ca; pH 5.5–6.0. The plants were cultured under the above-described condition.

One-week-old plants were divided into two groups. A non-acclimated plant group (NAP) which was irrigated with tap water, and an acclimated plant group (AP) irrigated four times with 20 mM NaCl at intervals of seven days. Five-week-old plants were used for salt treatment.

The experiments were performed on fully expanded third and fourth leaves (from the bottom). The leaves were harvested in the middle of the 16 h light period. Immediately after harvesting, the leaves were homogenized in a cold mortar. The activities of enzymes as well as lipid peroxides, α-tocopherol, carbonyl protein group and protein levels were examined after 24 (24 h) and 72 h (72 h) after moderate (100 mM) and severe (150 mM) stress application.

The analyses were carried out in leaves from two groups of cucumber plants:

The first group-non-acclimated plants:
  Non-acclimated–unstressed (NAP, control for non-acclimated plants subjected to salt stress);
  Non-acclimated stressed with 100 mM NaCl (NAP-100);
  Non-acclimated stressed with 150 mM NaCl (NAP-150).
The second group–acclimated plants:
  Acclimated–unstressed (AP, control for the acclimated plants subjected to salt stress);
  Acclimated stressed with 100 mM NaCl (AP-100);
  Acclimated stressed with 150 mM NaCl (AP-150).

### 2.2. Methods

#### 2.2.1. Enzymes Extract Preparation

To determine P5CS, P5CR, PDH activities and protein content, 500 mg of fresh leaves were homogenized at 4 °C in 2.5 cm^3^ of 50 mM Tris-HCl buffer (pH 7.6) containing 1 mM MgCl_2_, 1 mM EDTA, 1 mM dithiothreitol, 10 mM β-mercaptoethanol and 0.5% polyvinylpyrrolidone. The homogenate was centrifuged at 20,000× *g* for 20 min at 4 °C. The supernatant was used to determine enzyme activity and protein content.

To determine the activities of antioxidant enzymes, lipid peroxides, α-tocopherol and protein level, fresh leaves (500 mg) were homogenized at 4 °C in 5 cm^3^ of 100 mM sodium phosphate buffer (pH 7.5) containing 1 mM ascorbic acid, 1 mM EDTA, 0.5 M NaCl, 1%. The homogenate was centrifuged at 20,000× *g* for 20 min at 4 °C. The supernatant was used to determine enzyme activity as well as lipid peroxides and protein content. Crude extracts were used for α-tocopherol level determination.

#### 2.2.2. Proline Metabolism Enzymes

P5CS activity was measured spectrophotometrically at 25 °C by monitoring the oxidation of NADPH (*ε* = 6.22 mM^−1^ cm^−1^) at 340 nm according to Garcia-Rios et al. [30]. The reaction mixture contained 100 mM Tris-HCl buffer (pH 7.2), 25 mM MgCl_2_, 75 mM Na-glutamate, 5 mM ATP, 0.4 mM NADPH and the enzyme extract. P5CS activity was expressed in units, each representing the amount of enzyme catalyzing the oxidation of 1 nmol NADPH (ε = 6.22 mM^−1^ cm^−1^) per minute and expressed in U mg^−1^ protein.

P5CR and PDH activities were detected according to Szoke et al. [31] and by Rena and Splittstoesser [32]. P5CR activity was assayed at 340 nm following the oxidation of NADH. The reaction mixture comprised 50 mM potassium phosphate buffer pH 7.2, 128 μM NADH, 400 μM D,L-P5C and the enzyme extract. A unit of enzyme activity was defined as the amount of enzyme needed to cause a decrease in absorbance by 1 nmol NADH (*ε* = 6.22 mM^−1^ cm^−1^) per minute and expressed in U mg^−1^ protein. PDH activity was assayed following the reduction of NAD^+^ at 340 nm at 32 °C. The assay mixture contained 100 mM Na_2_CO_3_-NaHCO_3_ buffer, pH 10.3, 10 mM NAD^+^, 20 mM L-proline and the enzyme extract, where proline was used as a starter. The activity was calculated using the extinction coefficient of ε = 6.22 mM^−1^ cm^−1^. PDH activity was expressed in units, each representing the amount of enzyme catalyzing the reduction of 1 nmol NAD^+^ per minute and expressed in U mg^−1^ protein.

#### 2.2.3. Antioxidant Enzymes

APX activity was measured as described by Nakano and Asada [33]. Enzyme activity was assayed following the oxidation of ascorbate to dehydroascorbate at 265 nm. The assay mixture contained 50 mM sodium phosphate buffer (pH 7.0), 0.25 mM ascorbic acid, 25 μM H_2_O_2_ and enzyme extract, where hydrogen peroxide was used as a starter. A blank consisting of an ascorbate reaction mixture without the enzyme extract was used to correct for nonenzymatic oxidation. The activity was calculated using the extinction coefficient of *ε* = 13.7 mM^−1^ cm^−1^. The enzyme activity was expressed in (μmol ascorbate min^−1^ mg^−1^ protein) U mg^−1^ protein.

CAT activity was measured spectrophotometrically according to Dhindsa et al. [34]. The reaction mixture contained 50 mM sodium phosphate buffer (pH 7.0), 15 mM H_2_O_2_ and the enzyme extract. H_2_O_2_ decomposition was measured at 240 nm. The activity was calculated using an extinction coefficient of *ε* = 45.2 M^−1^ cm^−1^. The enzyme activity was expressed in (mmol H_2_O_2_ min^−1^ mg^−1^ protein) U mg-^1^ protein.

GST activity was determined with 1-chloro-2,4-dinitrobenzene, due to 1-chloro-2,4-dinitrobenzene-glutathione conjugation, according to Habig et al. [35]. The reaction solution contained 100 mM potassium phosphate buffer (pH 6.25), 0.75 mM CDNB, 30 mM glutathione and the enzyme extract. The activity was calculated from the increase in absorbance at 340 nm for 1 min when the extinction coefficient was 9.6 mM^−1^ cm^−1.^ The enzyme activity was expressed in (μmol 2,4-dinitrophenyl-*S*-glutathione min^−1^ mg^−1^ protein) U mg^−1^ protein.

GPX activity was determined according to Hopkins and Tudhope [36] with *t*-butyl hydroperoxide as a substrate. The reaction mixture comprised 50 mM potassium phosphate buffer (pH 7.0), 2 mM EDTA, 0.28 μM NADPH, 0.13 μM glutathione, 0.16 U glutathione reductase, 0.073 μM *t*-butyl hydroperoxide and the enzyme extract. Oxidation of NADPH+H^+^ was recorded at 340 nm for 6 min, and the activity was calculated using the extinction coefficient of *ε* = 6.22 mM^−1^ cm^−1^. The enzyme activity was expressed in (μmol NADPH min^−1^ mg^−1^ protein) U mg^−1^ protein.

#### 2.2.4. Determination of Lipid Peroxides

The concentration of lipid peroxides was estimated spectrofluorometrically according to Yagi [37] by measuring the content of 2-thiobarbituric acid reactive substances (TBARS). The concentration of lipid peroxides was calculated in terms of 1,1,3,3-tetrethoxypropane, which was used as a standard and expressed in nmol mg^−1^ protein.

#### 2.2.5. Protein Carbonyl Groups Determination

Carbonyl group (CO) content was estimated according to Levine et al. [38]. The leaves were homogenized in 0.05 M potassium–phosphate buffer (pH 7.5), containing 1 mM EDTA, 2 mM dithiothreitol, 0.2% (*v/v*) Triton X-100 and phenylmethane sulfonyl fluoride. The homogenates were centrifuged at 20,000× *g* for 20 min, and supernatants were used for the determinations. Samples containing at least 0.5 mg proteins were incubated with 1% (*w/v*) streptomycin sulfate for 20 min to remove the nucleic acids. After centrifuging at 2000× *g*, the supernatants (200 μL) were mixed with 300 μL of 10 mM 2,4-dinitrophenylhydrazine in 2 M HCl. The blank was incubated in 2 M HCl. After one-hour incubation at room temperature, proteins were precipitated with 10% (*w/v*) trichloroacetic acid. The pellets were washed three times with ethanol:ethyl acetate (1:1). Finally, the pellets were dissolved in 6 M guanidine hydrochloride in 20 mM potassium phosphate buffer (pH 2.3) at 37 °C. Absorbance was measured at 370 nm. Protein recovery was estimated for each sample by measuring the absorption at 280 nm. Carbonyl group content was calculated using the molar absorption coefficient for aliphatic hydrazone of *ε* = 22,000 M^−1^ cm^−1^. The concentration of CO was expressed in nmol mg^−1^ protein.

#### 2.2.6. Determination of α-Tocopherol

Tocopherol content was assayed according to Taylor et al. [39]. After saponification of the sample with 10 N KOH in the presence of 1.42 M ascorbic acid, α-tocopherol was extracted to *n*-hexane. Fluorescence of the organic layer was measured at 280 nm (excitation) and 310 nm (emission). The concentration of α-tocopherol was calculated in terms of α-tocopherol, which was used as a standard and expressed in μmol mg^−1^ protein.

#### 2.2.7. Protein Determination

Protein contents were measured as described by Bradford [40] using BSA as a standard.

#### 2.2.8. Statistical Analysis

The significance of differences between mean values was determined by the nonparametric Mann–Whitney Rank-Sum Test using Statistica 13 software. Differences at *p* < 0.05 were considered significant. Data are given as mean values ± standard deviation. Each data point is the mean of four independent replicates (n = 4). Each replicate consisted of three plants.

## 3. Results

### 3.1. The Effect of Salt Stress on the Antioxidant System

**Figure 1:**
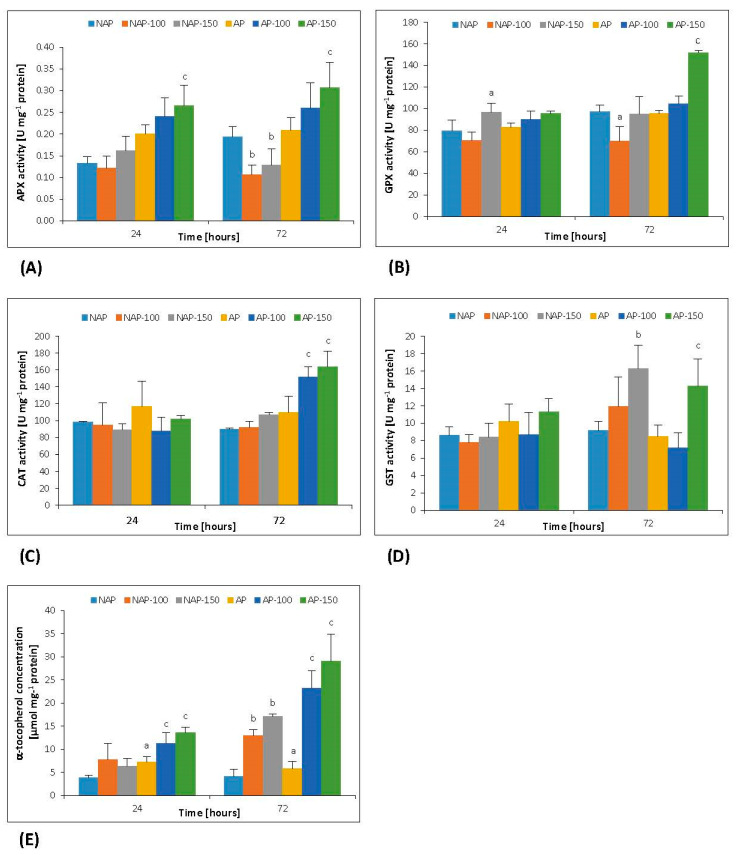
Changes in ascorbate peroxidase (APX) (**A**), glutathione peroxidase (GPX) (**B**), catalase (CAT) (**C**), glutathione *S*-transferase (GST) (**D**) activities and α-tocopherol (**E**) concentration in cucumber leaves in plants treated with 100 and 150 mM NaCl: non-acclimated (NAP) and acclimated (AP) to salinization. Bars represent SD of means, n = 4. a—indicates a significant difference between NAP and AP; b—indicates a significant difference between NAP, NAP-100 and NAP-150; c—indicates a significant difference between AP, AP-100 and AP-150.

In the non-acclimated plants, moderate and severe salt stress after 72 h caused a decrease in APX activity by 45% (NAP-100) and 33% (NAP-150), as compared to the respective controls (NAP) (Figure 1A). Severe stress application induced significant changes in APX activity in the acclimated plant (AP-150) after 24 and 72 h, being significantly higher, i.e., by about 30–45%, in the stressed group than its respective non-stressed group (AP).

Regarding moderate stress, GPX activity in NAP-100 decreased significantly by 25% compared to NAP after 72 h (Figure 1B). For more severe stress, a 21% increase of GPX activity was observed in NAP-150 compared to NAP after only 24 h. Further exposure to this level of stress did not change GPX activity compared to the respective control (NAP). In the acclimated plants, GPX activity increased significantly by 59% higher after 72 h following severe salt stress application, while no increase was observed following moderate stress (Figure 1B).

No change in CAT activity was observed in NAP-100 or NAP-150 throughout the experiment (Figure 1C). Significant increases in CAT activity were observed only after 72 h in the acclimated plants, i.e., by 38% (AP-100) and 49% (AP-150) compared with AP.

Moderate salt stress did not significantly influence GST activity in either group of cucumber plants (Figure 1D). Severe stress application induced significant changes in GST activity after 72 h: it was about 80% higher in both NAP-150 and AP-150 than in the respective non-stressed groups.

The process of acclimation significantly influenced the constitutive α-tocopherol concentration in the studied variants. The level was 40–80% higher in AP than NAP (Figure 1E). Following moderate and severe salt stress, the α-tocopherol levels increased significantly by 200% in NAP-100 and 300% in NAP-150 after 72 h, in comparison to NAP. These values were about 60% higher in AP-100 and 87% higher in AP-150 compared to AP after 24 h, and 300% and 400% higher after 72 h compared to AP.

### 3.2. The Effect of Salt Stress on the Oxidative Stress Markers Level

**Figure 2:**
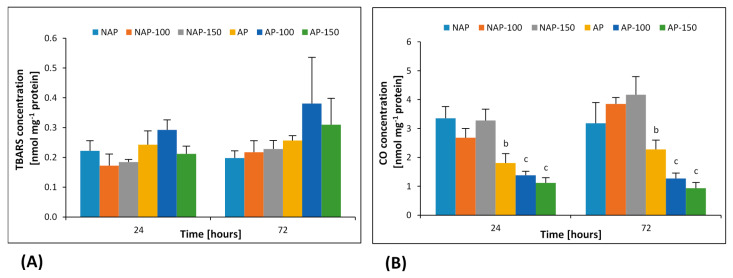
Changes in 2-thiobarbituric acid reactive substances (TBARS) (**A**) and protein carbonyl group (CO) (**B**) concentrations in cucumber leaves in plants treated with 100 and 150 mM NaCl: non-acclimated (NAP) and acclimated (AP) to salinization. Bars represent SD of means, n = 4. a—indicates a significant difference between NAP and AP; b—indicates a significant difference between NAP, NAP-100 and NAP-150; c—indicates a significant difference between AP, AP-100 and AP-150.

No change in TBARS level was observed in any examined group (Figure 2A).

The acclimation process significantly influenced carbonyl protein group content (Figure 2B). The constitutive CO level was 30–47% lower in AP than in NAP. In the acclimated plants, compared to AP, CO content significantly decreased by 23% in AP-100 and 38% in AP-150 after 24 h; it also decreased by 43% (AP-100) and 66% (AP-150) after 72 h.

### 3.3. The Effect of Salt Stress on the Proline Metabolism Enzymes Activity.

**Figure 3:**
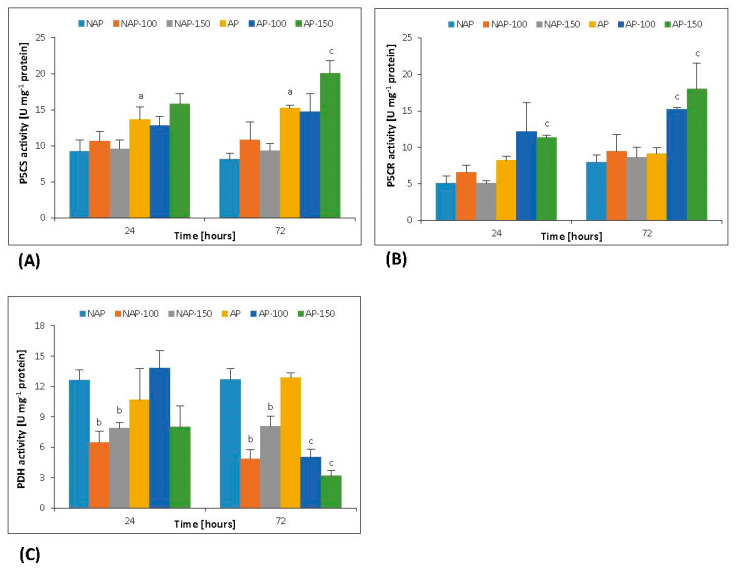
Changes in pyrroline-5-carboxylate synthetase (P5CS) (**A**), pyrroline-5-carboxylate reductase (P5CR) (**B**) and proline dehydrogenase (PDH) (**C**) activities in cucumber leaves in non-acclimated (NAP) and acclimated (AP) plants treated with 100 and 150 mM NaCl. Bars represent SD of means, n = 4. a—indicates a significant difference between NAP and AP; b—indicates a significant difference between NAP, NAP-100 and NAP-150; c—indicates a significant difference between AP, AP-100 and AP-150.

The process of acclimation significantly influenced the constitutive P5CS activity: it was 65% higher in AP than in NAP (Figure 3A). No change in P5CS activity was observed in the non-acclimated plants after moderate (NAP-100) or severe (NAP-150) stress treatment. However, in the acclimated plants, P5CS activity significantly increased by 32% in AP-150 after 72 h compared to AP.

In response to moderate stress, after 72 h, P5CR activity was 67% higher in AP-100 compared to AP. In the acclimated plants, severe stress significantly increased P5CR activity by 38% after 24 h and 98% after 72 h compared to AP (Figure 3B).

In response to moderate stress, PDH activity in the non-acclimated plants (NAP-100) decreased by 50% and 62% compared to NAP after 24 and 72 h, respectively. In the NAP-150 plants, PDH activity was about 37% and 55% lower after 24 and 72 h, respectively, than in NAP. Similarly, in the plants acclimated to stress, PDH activity decreased by 61% in AP-100 and 75% in AP-150, compared to AP after 72 h (Figure 3C).

## 4. Discussion

Our present findings extend our previous studies [27] aimed at understanding the relationship between tolerance to salt stress in plant acclimation and changes in primary nitrate and carbon metabolism. The salt-acclimated plants demonstrated i) higher activities of enzymes involved in glutamic acid production and ii) higher activities of enzymes in the oxidative pentose phosphate pathway, which could be attributed to high demand for glutamic acid and NADPH, respectively. The high demand for glutamic acid reflected a greater need for proline, an effective osmoprotectant. Similarly, the high demand for NADPH may be related to its participation in many metabolic processes where it is a cofactor central to metabolism, e.g., antioxidant system, proline synthesis.

To protect cells against the toxic effects of ROS, plants use a complex antioxidant system composed of enzymes and nonprotein compounds. Previous studies have examined the activity of ascorbate peroxidase in plants subjected to biotic and abiotic stresses, including pathogen–plant interactions [41,42]. Their findings partially confirm our present ones regarding the activity of APX under salt stress: its activity was found to increase only in acclimated plants, i.e., those subjected to prior salt stress (Figure 1A). In the non-acclimated ones, this activity either did not change or decrease in relation to the respective control, and the nature of these changes depended on the duration of stress and its intensity. The varied dynamics of APX activity observed in response to the applied stress may also be related, e.g., to the availability of ascorbate, one of the substrates needed for its reaction [42,43]. Mittov et al. [44,45] observed a decrease in the reduced form of ascorbate in organelles within the leaves and roots of tomato (*Lycopersicon esculentum* Mill.) characterized as a salt-sensitive species, and that this decrease was not accompanied by any changes in APX activity. However, a shift in the balance between ascorbate/dehydroascorbate towards dehydroascorbate was observed. This may be related to the antioxidant properties of ascorbic acid, which is directly involved in the removal of ROS, as well as the regeneration of tocopherol [43,46]. The increase in APX activity observed in the leaves of plants acclimated to stress in response to salt stress may be connected with increased stress tolerance (Figure 1A). Hernandez et al. [47] and Mittova et al. [44,45] report an increase in APX in the leaves of salt-tolerant pea (*Pisum sativum* L.) cultivar [47] and in tomato (*Lycopersicon pennellii* Corr.) characterized as a wild salt-tolerant species [44,45].

Many studies indicate a positive correlation between the activity of catalase and the degree of stress intensity [22]. An increase in CAT activity proportional to applied NaCl concentration was observed, e.g., in white mulberry (*Morus alba* L.) in response to salinity [48]. The fact that CAT activity increased in the acclimated plants, but not the non-acclimated ones (Figure 1C) under salt stress indicates that acclimation may cause changes in metabolic reactions taking place in peroxisomes and that this may be related to the activation of processes preventing pro-oxidative changes under stressful conditions. An increase in CAT in leaves peroxisomes in wild salt-tolerant species of tomato was reported by Mittova et al. [44]. The increase in CAT activity observed during salt stress in the present study is confirmed in studies carried out on *Cucumis melo* var. Inodorus [49] found that seedlings whose seeds were conditioned in sodium chloride solution with EC = 18 dS m^−1^ demonstrated the greatest increase in CAT activity in response to salt stress.

The presence of carbonyl groups in proteins caused by oxidation is the most commonly determined marker of oxidative stress in aerobic organisms. Protein carbonyl groups are particularly common in organelles subjected to increased ROS generation [15]. The occurrence of protein carbonylation taking place in response to environmental stress may have a signaling function. It has been suggested that carbonylated proteins outside the mitochondria may function as secondary messengers [15,16]. The acclimated plants in the present study demonstrated a decrease in protein carbonyl level (Figure 2B); this may be related to the presence of compounds with osmoprotective functions such as proline. Similarly, Hoque et al. [50] report an exogenous application of proline or glycine betaine resulted in a reduction of protein carbonylation in suspension-cultured cells of *Nicotiana tabacum* L., cv. BY-2 (Bright Yellow-2). The fact that lower levels of protein carbonylation were observed in acclimated plants after the application of salt stress may also be related to the degradation of these compounds by proteases. Pena et al. [51] found the activity of proteolytic enzymes to be elevated in response to oxidative stress initiated by the presence of heavy metals. Our results show a low constitutive concentration of protein carbonyl groups in the acclimated plants, which may indicate the activity of a well-functioning proteolysis mechanism, together with a well-functioning ROS removal system.

Another marker of oxidative stress is the presence of lipid peroxidation products, which react with 2-thiobarbituric acid [17]. In both studied groups of cucumber plants, no significant changes in lipid peroxides content were observed throughout the experiment in response to either dose (Figure 2B). The obtained results are similar to those published by Cai-Hong et al. [52], who found a slight decrease in TBARS concentration in the leaves of *Sueda salsa* L. subjected to salt stress. These findings may be indicative of a highly effective system of hydrophobic antioxidant compounds used to remove lipid peroxidation products. Tocopherols are strongly hydrophobic antioxidants [46]. However, the increases in α-tocopherol concentration (Figure 1E) and glutathione-dependent enzyme activity (Figure 1B,D) in response to stress appear to inhibit TBARS growth. Cellular tocopherol levels vary between individual plant organs and change according to the plant development stage and the presence of stress factors. As in the case of hydrophilic antioxidants, the concentration of tocopherols (mainly α-tocopherol) increases in response to environmental stresses [46]. Keleş and Öncel [53] report that salinity induces the synthesis and accumulation of α-tocopherol in wheat seedlings. Skłodowska et al. [54] also note that salt stress induced an increase in the concentration of α-tocopherol in tomato chloroplasts, with the extent of this induction correlating with the concentration of NaCl and the time of exposure of plants to stress. These results were confirmed in our study (Figure 1E), where higher intensities and duration of salt stress were accompanied by a progressive increase in α-tocopherol concentration in both groups of cucumbers. This may explain the lack of changes observed in TBARS concentration after stress.

Biotic and abiotic stress also has been found to result in an increase in GPX activity [55], as observed in chloroplasts and whole leaves of tomatoes subjected to salt stress [54]. In the present study, the increase in GPX activity in the acclimated plants was found to depend on the level of applied NaCl stress (Figure 1B). These observations are consistent with studies conducted on *P. sativum* by Hernandez et al. [47], who found that levels of phospholipid-hydroperoxide GPX transcripts increased in the leaves of tolerant cultivar, but not insensitive ones. The increase in GST and GPX activities observed during salt stress in the present study, particularly in the acclimated plants, may suggest that these glutathione-dependent enzymes participate in increasing tolerance. Roxas et al. [56,57] found that genetically modified tobacco seedlings demonstrating GPX and GST overexpression tolerated salt stress better than the wild variety, showed normal growth and development under high soil salinity, suggesting that increased activity of glutathione-dependent enzymes may be associated with increased tolerance to salt stress [56,57].

A common reaction to salt stress is an increase in the synthesis and accumulation of proline. Many studies clearly indicate that proline plays a role in the protection of structural components in cells and in the enzymes involved in the antioxidant defense [58]. Tobacco BY-2 cell suspensions culture subjected to exogenous application of proline demonstrated significantly lower ROS and TBARS concentrations, alleviated the reduction in peroxidase and CAT activity, and less growth inhibition, in response to salt stress compared to untreated cultures [50,59]. In another study based on an identical experimental model, tobacco BY-2 cell cultures treated, e.g., with proline, demonstrated reduction of NaCl-induced cell death via decreasing level of ROS accumulation and lipid peroxidation as well as improvement of membrane integrity [60]. In addition, in a study based on acclimated cucumber suspension cell cultures, free proline level was found to correlate with high vigor during salinization [61]. In our previous work [27], we used cucumber plants acclimated to salt stress; we reported that osmoprotectants such as proline, glucose and sucrose levels in cucumber leave tissues increased in response to salt stress, especially in the late phase of the experiment what made that the acclimated plants were in better condition following salt stress than the non-acclimated ones

Generally, the increase in proline concentration is directly related to the activity of the enzymes involved in its synthesis and degradation. In plant cells, the enzyme involved in the conversion of glutamate to pyrroline-5-carboxylic acid, a stage in proline synthesis, is pyrroline-5-carboxylic acid synthetase [24,29]. In the present study, generally, salt stress did not cause significant changes in P5CS activity in either examined group (Figure 3A), but the acclimation process increased the constitutive P5CS activity in AP by 50%. Similarly, Wang et al. [62] report no change in P5CS activity in *Triticum estivum* seedlings subjected to 150 and 300 mM NaCl, but an increase in P5CR activity in response to 300 mM NaCl. The lack of changes in P5CS activity observed in non-acclimated plants following salt stress may be related to the fact that P5CS activity is tightly regulated by proline level, with increased concentrations of proline causing enzyme inactivation by binding to its γ-GK domain [24,29]. Our results show that the acclimated plants demonstrated a greater increase in P5CR activity in response to salt stress (Figure 3B). The lack of changes in P5CR activity observed in the non-acclimated plants may have been due to a lack of change in P5CS activity. On the other hand, the increase in P5CR activity seen in the acclimated plants in response to salt stress may reflect the activity of a different P5C synthesis pathway than that involving P5CS [29]. The accumulation of proline in plants following stress is not always associated with increased activity by its synthesis enzymes [28,29]; in fact, numerous studies have found the activity of enzymes in the proline oxidation pathway (i.e., proline degradation) to be significantly reduced during salt stress. This is particularly true for proline dehydrogenase, the first enzyme in the proline degradation pathway, which oxidizes this compound to P5C [28]. Salt stress was found to cause a decrease in PDH activity in the non-acclimated and acclimated plants (Figure 3C). Interestingly, although this decrease occurred in both examined time periods in the non-acclimated group, it was observed mainly in the late period in the acclimated group. A similar phenomenon was observed in *Triticum durum* Desf. leaves, where PDH activity progressively decreased with the duration of exposure to salt stress [63]. Our previous results showed [27] that the concentration of proline progressively increased with the time of exposure to NaCl in both acclimated and non-acclimated groups; they also indicate this occurred together with a lack of, or a slight increase in, the activity of proline anabolism enzymes (Figure 3A,B) it can be assumed that low activity of proline dehydrogenase plays a significant role in proline accumulation. In the present study, both studied groups of cucumber plants demonstrated a decrease in PDH activity in response to salinity. This may result from the properties of this enzyme. PDH activity is controlled by the amount of substrate, so increasing proline concentration should activate PDH [24,64]. However, PDH activity is inhibited under unfavorable environmental conditions, despite high proline concentrations, whereas the removal of stress factors results in immediate activation of the expression of genes encoding *PDH* [24]. Peng et al. [64] showed that treatment with exogenous proline led to an increase in *PDH* gene expression in *Arabidopsis thaliana* seedlings and that this increase was inhibited by the application of salt stress. In the present study, following salt stress treatment, PDH activity was found to fall earlier in the non-acclimated plants than in the acclimated ones; this suggests that the potential osmotic decreases faster in the former than in the latter. Importantly, despite the significant increase in proline concentration caused by the inhibition of PDH activity, P5CS activity was not found to be suppressed for all the examined groups of plants. The proline accumulated during stress can be used for the synthesis of proline betaine, whose osmoprotective properties are more potent than those of proline. An increase in proline betaine concentration causes a decrease in the level of free proline, which leads to inhibition of proline oxidation by PDH [24].

## 5. Conclusions

Our results partially confirm the commonly accepted hypothesis that the degree of tolerance to salt stress is closely related to the effectiveness of the antioxidant defense of the cell and that this may, in turn, be reflected in an increase in the activity of antioxidant enzymes or their high constitutive activity. Apart from α-tocopherol, none of the tested antioxidants demonstrated increased constitutive activity in the acclimated plants; however, the antioxidant enzyme activity grew in response to salt stress. The differences observed in response to salt stress between the acclimated and non-acclimated plants may suggest a relationship between increased tolerance to salinity stress and the increased activity of APX, CAT, glutathione-dependent enzymes and high concentration of α-tocopherol as well as decreased activity of proline catabolism enzyme. We can suggest that certain metabolic changes occur during acclimation to stress, allowing more efficient activation of defense reactions to stress. The efficient removal of protein and lipid oxidation products demonstrated in the acclimated plants, as well as the ROS inducing these processes, may be associated with greater resistance to oxidative stress.

## Data Availability

The data presented in this study are available on request from the corresponding author.

## References

[1] Van Zelm E., Zhang Y., Testerink C. (2020). Salt tolerance mechanisms of plants. Annu. Rev. Plant Biol..

[2] Munns R., Tester M. (2008). Mechanisms of salinity tolerance. Annu. Rev. Plant Biol..

[3] FAO Soils Portal. http://www.fao.org/soils-portal/soil-management/management-of-some-problem-soils/en/.

[4] Safdar H., Amin A., Shafiq Y., Ali A., Yasin R. (2019). A review: Impact of salinity on plant growth. Nat. Sci..

[5] Flowers T.J., Munns R., Colmer T.D. (2015). Sodium chloride toxicity and the cellular basis of salt tolerance in halophytes. Ann. Bot..

[6] Parida A.K., Das A.B. (2005). Salt tolerance and salinity effects on plants: A review. Ecotoxicol. Environ. Saf..

[7] Acosta-Motos J.A., Ortuño M.F., Bernal-Vicente A., Diaz-Vivancos P., Sanchez-Blanco M.J., Hernandez J.A. (2017). Plant responses to salt stress: Adaptive mechanisms. Agronomy.

[8] Ashraf M., Harris P.J.C. (2004). Potential biochemical indicators of salinity tolerance in plants. Plant Sci..

[9] Parihar P., Singh S., Singh R., Singh V.P., Prasad S.M. (2015). Effect of salinity stress on plants and its tolerance strategies: A review. Environ. Sci. Pollut. Res. Int..

[10] Strogonov B.P., Poljakoff-Mayber A., Meyer A.A. (1964). Practical Means for Increasing Salt Tolerance of Plants as Related to Type of Salinity in the Soil. Physiological Basis of Salt Tolerance of Plants.

[11] Hossain Z., Mandal A.K.A., Datta S.K., Biswas A.K. (2007). Development of NaCl-tolerant line in *Chrysanthemum morifolium* Ramat. through shoot organogenesis of selected callus line. J. Biotechnol..

[12] Queirós F., Fidalgo F., Santos I., Salema R. (2007). In vitro selection of salt tolerant cell lines in *Solanum tuberosum* L.. Biol. Plant..

[13] Duhan S., Kumari A., Lal M., Sheokand S., Hasanuzzaman M., Fotopoulos V., Nahar K., Fujita M. (2019). Oxidative Stress and Antioxidant Defense under Combined Waterlogging and Salinity Stresses. Reactive Oxygen, Nitrogen and Sulfur Species in Plants: Production, Metabolism, Signaling and Defense Mechanisms.

[14] Talaat N.B., Hasanuzzaman M., Fotopoulos V., Nahar K., Fujita M. (2019). Role of Reactive Oxygen Species Signaling in Plant Growth and Development. Reactive Oxygen, Nitrogen and Sulfur Species in Plants: Production, Metabolism, Signaling and Defense Mechanisms.

[15] Møller I.M., Jensen P.E., Hansson A. (2007). Oxidative modification to cellular components in plants. Annu. Rev. Plant Biol..

[16] Sweetlove L.J., Møller I.M., Jacquot J.P. (2009). Oxidation of Proteins in Plants-Mechanisms and Consequences. Advances in Biological Research. Oxidative Stress and Redox Regulation in Plants.

[17] Soltabayeva A., Ongaltay A., Omondi J.O., Srivastava S. (2021). Morphological, physiological and molecular markers for salt-stressed. Plants.

[18] Francoz E., Ranocha P., Nguyen-Kim H., Jamet E., Burlat V., Dunand C.H. (2015). Roles of cell wall peroxidases in plant development. Phytochemistry.

[19] Lee B.-R., Kim K.-Y., Jung W.-J., Avice J.-C.H., Ourry A., Kim T.-H. (2007). Peroxidases and lignification in relation to the intensity of water-deficit stress in white clover (*Trifolium repens* L.). J. Exp. Bot..

[20] Bolwell G.P., Wojtaszek P. (1997). Mechanisms for the generation of reactive oxygen species in plant defense—A broad perspective. Physiol. Mol. Plant. Pathol..

[21] Pandey V.P., Awasthi M., Singh S., Tiwari S., Dwivedi U.N. (2017). A comprehensive review on function and application of plant peroxidases. Anal. Biochem..

[22] Mhamdi A., Queval G., Chaouch S., Vanderauwera S., van Breusegem F., Noctor G. (2010). Catalase function in plants: A focus on Arabidopsis mutants as stress-mimic models. J. Exp. Bot..

[23] Marrs K.A. (1996). The functions and regulation of glutathione *S*-transferases in Plants. Annu. Rev. Plant Physiol. Plant Mol. Biol..

[24] Rhodes D., Verslues P.E., Sharp R.E., Singh B.K. (1999). Role of Amino Acids in Abiotic Stress Resistance. Plant Amino Acids. Biochemistry and Biotechnology.

[25] Signorelli S., Arellano J.B., Melø T.B., Borsani O., Monza J. (2013). Proline does not quench singlet oxygen: Evidence to reconsider its protective role in plants. Plant Physiol. Biochem..

[26] Signorelli S., Coitiño E.L., Borsani O., Monza J. (2014). Molecular mechanisms for the reaction between OH radicals and proline: Insights on the role as reactive oxygen species scavenger in plant stress. J. Phys. Chem. B.

[27] Naliwajski M.R., Skłodowska M. (2018). The relationship between carbon and nitrogen metabolism in cucumber leaves acclimated to salt stress. PeerJ.

[28] Verma D.P., Zhang C.-S., Singh B.K. (1999). Regulation of Proline and Arginine Biosynthesis in Plants. Plant Amino Acids. Biochemistry and Biotechnology.

[29] Szabados L., Savoure A. (2010). Proline: A multifunctional amino acid. Trends Plant Sci..

[30] Garcia-Rios M., Fujita T., LaRosa P.C., Locy R.D., Clithero J.M., Bressan R.A., Csonka L.N. (1997). Cloning of a polycistronic cDNA from tomato encoding γ-glutamyl kinase and γ-glutamyl phosphate reductase. Proc. Natl. Acad. Sci. USA.

[31] Szoke A., Miao G., Hong Z., Verma D.P.S. (1992). Subcellular location of Δ^1^-pyrroline-5-carboxylate reductase in root/nodule and leaf of soybean. Plant Physiol..

[32] Rena A.B., Splittstoesser W.E. (1975). Proline dehydrogenase and pyrroline-5-carboxylate reductase from pumpkin cotyledons. Phytochemistry.

[33] Nakano Y., Asada K. (1981). Hydrogen peroxide is scavenged by ascorbate-specific peroxidase in spinach chloroplasts. Plant Cell Physiol..

[34] Dhindsa R.S., Plumb-Dhindsa P., Thorpe T.A. (1981). Leaf senescence: Correlated with increased levels of membrane permeability and lipid peroxidation, and decreased levels of superoxide dismutase and catalase. J. Exp. Bot..

[35] Habig W.H., Pabst M.J., Jakoby W.B. (1974). Glutathione *S*-transferase. The first enzymatic step in mercaptane acid formation. J. Biol. Chem..

[36] Hopkins J., Tudhope G.R. (1973). Glutathione peroxidase in human red cells in health and disease. Br. J. Haematol..

[37] Yagi K., Yagi K. (1982). Assay for Serum Lipid Peroxide Level Its Clinical Significance. Lipid Peroxides in Biology and Medicine.

[38] Levine R.L., Garland D., Oliver C.N., Amici A., Climent I., Lenz A.G., Ahn B.W., Shaltiel S., Stadtman E.R. (1990). Determination of carbonyl content in oxidatively modified proteins. Methods Enzymol..

[39] Taylor S.L., Lamden M.P., Tappel A.L. (1976). Sensitive fluorometric method for tissue tocopherol analysis. Lipids.

[40] Bradford M.M. (1976). A rapid and sensitive method for the quantitation of microgram quantities of protein utilizing the principle of protein-dye binding. Anal. Biochem..

[41] Foyer C.H., Noctor G. (2005). Oxidant and antioxidant signaling in plants: A re-evaluation of the concept of oxidative stress in a physiological context. Plant Cell Environ..

[42] Sakhno L.O., Yemets A.I., Blume B.Y., Hasanuzzaman M., Fotopoulos V., Nahar K., Fujita M. (2019). The Role of Ascorbate-Glutathione Pathway in Reactive Oxygen Species Balance under Abiotic Stresses. Reactive Oxygen, Nitrogen and Sulfur Species in Plants: Production, Metabolism, Signaling and Defense Mechanisms.

[43] Akram N.A., Shafiq F., Ashraf M. (2017). Ascorbic acid—A potential oxidant scavenger and its role in plant development and abiotic stress tolerance. Front. Plant Sci..

[44] Mittova V., Tal M., Volkovita M., Guy M. (2002). Up-regulation of the mitochondrial and peroxisomal antioxidative systems in response to salt-induced oxidative stress in the wild salt-tolerant tomato species. Plant Cell Environ..

[45] Mittova V., Guy M., Tal M., Volkovita M. (2002). Response of the cultivated tomato and its wild salt-tolerant *Lycopersicon pennellii* to salt-dependent oxidative stress: Increased activities of antioxidant enzymes in root plastids. Free Radic. Res..

[46] Munné-Bosch S. (2005). The role of α–tocopherol in plant stress tolerance. J. Plant Physiol..

[47] Hernandez J.A., Jimenez A., Mullineaux P., Sevilla F. (2000). Tolerance of pea (*Pisum sativum* L.) to long-term salt stress is associated with induction of antioxidant defences. Plant Cell Environ..

[48] Sudhakar C., Lakshmi A., Giridarakumar S. (2001). Changes in antioxidant enzyme efficacy in two high yielding genotypes of mulberry (*Morus alba* L.) under NaCl salinity. Plant Sci..

[49] Sivritepe N., Sivritepe H.Ö., Türkan I., Bor M., Özdemir F. (2008). NaCl pre-treatment mediate salt adaptation in melon plants through antioxidative system. Seed Sci. Technol..

[50] Hoque M.A., Banu M.N.A., Nakamura Y., Shimoishi Y., Murata Y. (2008). Proline and glycinebetaine enhanced antioxidant defense and mathylglyoxal detoxification systems and reduced NaCl-induced damage in cultured tobacco cells. J. Plant Physiol..

[51] Pena L.B., Pausini L.A., Tomaro M.L., Gallego S.M. (2006). Proteolytic system in sunflower (*Helianthus annuus* L.) leaves under cadmium stress. Plant Sci..

[52] Cai-Hong P., Su-Jun Z., Zhi-Zhong G., Bao-Shan W. (2005). NaCl treatment markedly enhances H_2_O_2_-scavenging system in leaves of halophyte *Suaeda salsa*. Physiol. Plant..

[53] Keleş Y., Öncel I. (2002). Response of antioxidative defence system to temperature and water stress combinations in wheat seedlings. Plant Sci..

[54] Skłodowska M., Gapińska M., Gajewska E., Gabara B. (2009). Tocopherol content and enzymatic antioxidant activities in chloroplasts from NaCl-stressed tomato plants. Acta Physiol. Plant..

[55] Banerjee A., Roychoudhury A., Hasanuzzaman M., Fotopoulos V., Nahar K., Fujita M. (2019). Role of Glutathione in Plant Abiotic Stress Tolerance. Reactive Oxygen, Nitrogen and Sulfur Species in Plants: Production, Metabolism, Signaling and Defense Mechanisms.

[56] Roxas V.P., Smith R.K., Allen E.R., Allen R.D. (1997). Overexpression of glutathione *S*-transferase/glutathione peroxidase enhances the growth of transgenic tobacco seedlings during stress. Nat. Biotech..

[57] Roxas V.P., Lodhi S.A., Garrett D.K., Mahan J.R., Allen R.D. (2000). Stress tolerance in transgenic tobacco seedlings that overexpress glutathione *S*-transferase/glutathione peroxidase. Plant Cell Physiol..

[58] Hasanuzzaman M., Nahar K., Fujita M., Ahmad P., Azooz M.M., Prasad M.N.V. (2013). Plant Response to Salt Stress and Role of Exogenous Protectants to Mitigate Salt-Induced Damages. Ecophysiology and Responses of Plants under Salt Stress.

[59] Hoque M.A.O.E., Banu M.N.A., Nakamura Y., Shimoishi Y., Murata Y. (2007). Exogenous proline mitigates the detrimental effects of salt stress more than the betaine by increasing antioxidant enzyme activities. J. Plant Physiol..

[60] Banu N.A., Hoque A., Watanabe-Sugimoto M., Matsuoka K., Nakamura Y., Shimoishi Y., Murata Y. (2009). Proline and glicinebetaine induce antioxidant defense gene expression and suppress cell death in cultured tobacco cells under salt stress. J. Plant Physiol..

[61] Naliwajski M.R., Skłodowska M. (2014). Proline and its metabolism enzymes in cucumber cell cultures during acclimation to salinity. Protoplasma.

[62] Wang Z.Q., Yuan Y.Z., Ou J.Q., Lin Q.H., Zhang C.F. (2007). Glutamine synthetase and glutamate dehydrogenase contribute differentially to proline accumulation in leaves of wheat (*Triticum aestivum*) seedlings exposed to different salinity. J. Plant Physiol..

[63] Mattioni C., Lacerenza N.G., Troccoli A., de Leonardis A.M., Di Fonzo N. (1997). Water and salt stress-induced alterations in proline metabolism of *Triticum durum* seedlings. Physiol. Plant..

[64] Peng Z., Lu Q., Verma D.P.S. (1996). Reciprocal regulation of Δ^1^-pyrroline-5-carboxylate synthetase and proline dehydrogenase genes controls proline levels during and after osmotic stress in plants. Mol. Gen. Genet..

